# Development and validation of a nomogram for suspected post-neurosurgical bacterial ventriculitis/meningitis

**DOI:** 10.3389/fmed.2026.1790954

**Published:** 2026-03-18

**Authors:** Min Ni, Yu-ying Yan, Song-yu Chen, Lei Li, Fang Huang, Qi-bing Huang, Min Zhou, Chen-rui Shen, Qi Liu, Liang Gao

**Affiliations:** 1 Department of Pharmacy, Shanghai Tenth People’s Hospital, School of Life Sciences and Technology,Tongji University, Shanghai, China; 2 Department of Neurosurgery, Shanghai Tenth People’s Hospital, Tongji University School of Medicine, Shanghai, China; 3 Department of Neurosurgery, Shanghai Donglei Brain Hospital, Shanghai, China; 4 Department of Emergency Neurosurgery, Qilu Hospital, Shandong University, Ji’nan, China; 5 Department of Neurosurgery, Bengbu First People’s Hospital, Bengbu, China

**Keywords:** hypoglycorrhachia, leukocytic pleocytosis, MIMIC-III/IV database, post-neurosurgical bacterial ventriculitis/meningitis, risk scoring system

## Abstract

**Objective:**

The diagnosis of post-neurosurgical bacterial ventriculitis/meningitis (BV/M) remains challenging, particularly in the patients with leukocytic pleocytosis and hypoglycorrhachia. This study aimed to establish a nomogram identifying the high-risk and low-risk post-neurosurgical BV/M and evaluate the utility of this risk scoring system.

**Methods:**

Adult patients with CSF leukocytes ≥ 100/mm^3^ and glucose ≤ 2.2 mmol/L experienced neurosurgical or invasive procedures in three hospitals were divided into training and validation cohort, patients from Medical Information Mart for Intensive Care (MIMIC)-III and MIMIC-IV were also used as validation cohort. Multivariate logistic regression was performed to identify independent predictors and establish a nomogram to predict the occurrence of BV/M.

**Results:**

Totally, 271 patients (150 confirmed BV/M and 121 confirmed non-BV/M) selected from 711 suspected post-neurosurgical BV/M patients with leukocytic pleocytosis and hypoglycorrhachia were used as training cohort. CSF glucose, CSF leukocytes, CSF erythrocytes, CSF neutrophil proportions, blood lymphocyte proportions, and external ventricular drainage filtered out from 20 easily available parameters were used as independent predictors to develop the nomogram for BV/M. The high discriminative power of this nomogram was assessed by two independent validation cohorts (84 and 58 patients respectively).

**Conclusion:**

The nomogram uses six easily available indexes to predict BV/M risk. High-risk patients should receive immediate antibiotics, increasing CSF examination frequency is recommended before antibiotic treatment in low-risk patients.

## Background

Post-neurosurgical bacterial ventriculitis/meningitis (BV/M) is a life-threatening disease after invasive neurosurgical procedures (1, 2). BV/M has an insidious onset and is likely to be caused by resistant microorganisms (3), which may lead patients to experience severe neurological sequelae, prolonged hospital stays, and high costs (4).

Early administration of appropriate antibiotics is a crucial intervention to reduce mortality in patients with BV/M and empirical therapy was often initiated when a patient met leukocytic pleocytosis and hypoglycorrhachia. However, in clinical practice, it was found that not all patients with leukocytic pleocytosis and hypoglycorrhachia need antibiotics in subsequent observations. Unnecessary use of antibiotics to patients without infection does not improve outcomes but exposes patients to antibiotic-associated adverse drug reactions. Therefore, successful administration of antibiotics depends on early diagnosis of BV/M.

Ruling out bacterial contamination, positive cerebrospinal fluid (CSF) etiological culture is the most powerful evidence for the antibiotic prescription. However, the sensitivity of culture is 60% for CSF, what is more, the positive results will be generally available at 24 h at least (5). Though advanced techniques in diagnostic microbiology and neuroradiology have expanded the etiological differential diagnosis of BV/M (6), it is still unavailable for patients in developing countries or districts to obtain advanced diagnostic methods. Therefore, clinicians prefer to seek abnormalities of CSF parameters and unusual clinical features, *etc.* to determine whether to initiate antibiotic treatment.

Classical abnormalities of CSF in suspected BV/M, mainly referred to the increase of leukocytes and decrease of glucose concentration in CSF (7), make the clinicians to initiate empirical antibiotic therapy before positive CSF etiological culture, which turned out to be overused during the follow-up (8). Therefore, if the clinicians are able to differentiate the high-risk and low-risk post-neurosurgical BV/M depending on the abnormalities of CSF prior to the results from CSF etiological culture, it will help them to treat the high-risk patients properly without delay (9) and reduce the overuse of antibiotics in low-risk patients. By using the easily accessible clinical and laboratory parameters, this study was designed to establish a nomogram to predict the risk of post-neurosurgical BV/M in patients with CSF leukocytes (above 100/mm^3^) and glucose value (below 2.2 mmol/L), and thus providing a possible tool for improving the rational use of antibiotics.

## Methods

### Study design and population

The training cohort was derived from neurosurgery departments of Shanghai Tenth People’s Hospital, Qilu Hospital of Shandong University, and Bengbu First People’s Hospital during the period from Jan 1, 2018 to Dec 31, 2021. The validation cohort-1 was derived from neurosurgery departments of the three same hospitals during the period from Jan 1, 2022 to Jun 30, 2023. All the enrolled patients experienced neurosurgical or invasive procedures. Besides, patients from Medical Information Mart for Intensive Care (MIMIC)-III and MIMIC-IV were used for external validation namded as vaildation cohort-2.

The inclusive criteria were as follows: (a) experienced CSF bacterial culture; (b) CSF leukocytes ≥ 100/mm^3^ and CSF glucose ≤ 2.2 mmol/L; (c) age ≥18 years. Patients who met the inclusive criteria were classified into 2 groups: confirmed BV/M and non-BV/M.

For the BV/M group, patients should meet the following criteria: (a) positive CSF etiological culture; (b) confirmed BV/M in the discharge diagnosis. Conditions of the non-BV/M group were as follows: (a) negative CSF etiological culture; (b) non confirmed BV/M in the admission diagnosis; (c) steadily decreased CSF leukocytes after reaching the peak; (d) no adding/ changing antibiotics or increasing dosage occurred 3 days before and after the highest CSF leukocyte counting. Because of missing values in MIMIC-III and MIMIC-IV, other criteria for the non-BV/M group were selected: (a) negative CSF etiological culture; (b) non confirmed BV/M in the admission diagnosis; (c) non antibiotic prescription from the day that the patients with leukocytic pleocytosis and hypoglycorrhachia until discharge.

### Data collection

The collected data from enrolled patients included age, sex, surgical site, admitting diagnosis, temperature, C-reactive protein (CRP), blood leukocytes, blood neutrophil proportions, blood lymphocyte proportions, blood procalcitonin (PCT), blood lactate, CSF leukocytes (CSF leukocytes = Actual detected values for CSF leukocytes - CSF erythrocytes/800) (10), CSF neutrophil proportions, CSF erythrocytes, CSF proteins, CSF glucose, the glasgow coma scale (GCS), external ventricular drainage (EVD), concurrent infection and transparency of CSF. Data were recorded on the day that the CSF leukocyte count reached the highest point. Missing values were replaced by the median value of the corresponding group.

### Statistical methods

All of the continuous variables were transformed into categorical variables, making the generated model simple, feasible, and more suitable for clinical application. The cut-off values for 20 variables were determined by the decision tree (11). All statistical analyses were conducted by using R version 4.2.1 software with rms, rpart, pROC, ggplot2 and dca packages. Count data were expressed as a rate.

Independent predictors were assessed by multivariate logistic regression with a *p* < 0.01 to avoid overfitting (12) and then recruited to develop the nomogram. A nomogram was constructed to predict the risk of BV/M. Draw a prediction line upwards to confirm the points obtained from the column chart. Calculate the sum of these points, find the location on the “total points” axis, and then get the corresponding possibility of BV/M from the bottom scale. The value of 0.5, a threshold commonly used to divide prediction probability, was employed to distinguish between low-risk and high-risk of BV/M (13).

The fitness of the nomogram was assessed by the Hosmer-Lemeshow. The accuracy of the nomogram was evaluated by the receiver operating characteristic (ROC) curve and the area under the ROC curve (AUC). The consistency was displayed by the calibration curve. The net benefit for the patients of the nomogram was estimated by the decision curve analysis (DCA).

## Results

### General characteristics

**Figure 1:**
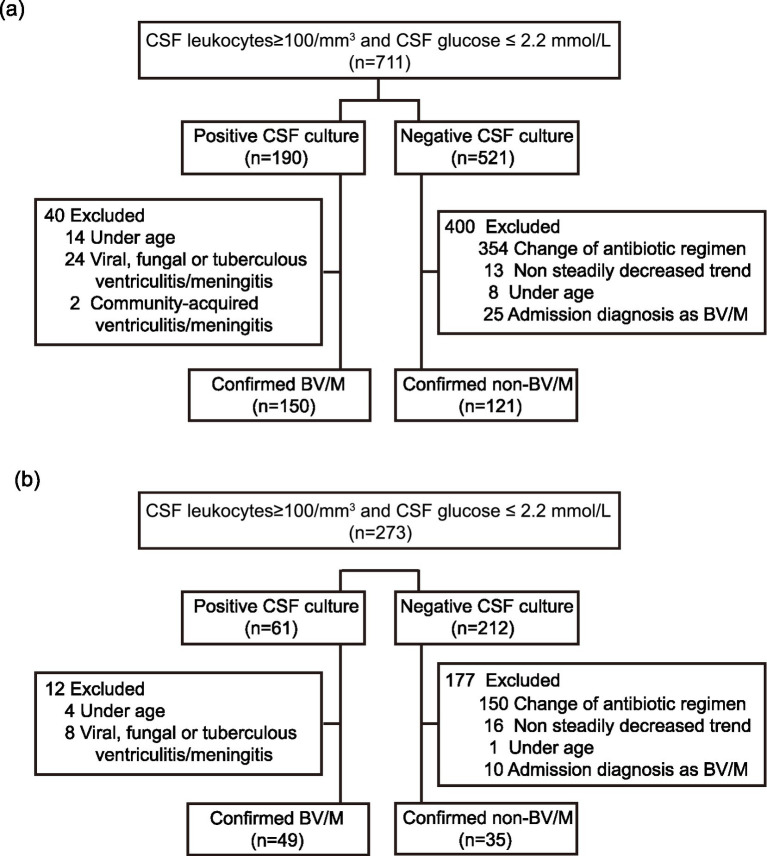
Flow chart for patient selection in the training and validation cohorts: **(a)** Training cohort; **(b)** validation cohort. CSF, Cerebrospinal fluid; BV/M, bacterial ventriculitis/meningitis.

Among 711 patients with CSF leukocytes (above 100/mm^3^) and glucose value (below 2.2 mmol/L) from the three hospitals during the period from Jan 1, 2018 to Dec 31, 2021, 190 patients were positive CSF etiological culture and 521 patients were negative CSF etiological culture. For patients with positive CSF etiological culture, 14 patients were under the age of 18, 24 were diagnosed as viral, fungal or tuberculous ventriculitis/meningitis, and 2 were diagnosed as community-acquired ventriculitis/meningitis. For patients with negative CSF etiological culture, 354 patients were added/changed antibiotics or increased the antibiotic dosage 3 days before and after the highest CSF leukocyte counting, 13 were non steadily decreased after reaching the peak of CSF leukocytes, 8 were u of 18, and 25 had admission diagnosis as BV/M. Finally, there were 150 confirmed BV/M patients and 121 confirmed non-BV/M patients ruled in training cohort (Figure 1a). The patient selection for the validation cohort-1 and cohort-2 (from MIMIC-III and MIMIC-IV databases) was shown in Figure 1b and Supplementary Figure S1, respectively. We searched the “d_labitems” table in the MIMIC-III /IV databases to identify patients who had undergone CSF laboratory testing, specifically those with available CSF glucose and CSF white blood cell count measurements. Among these patients, we reviewed the laboratory values and selected 248 patients who met our predefined inclusion criteria: CSF leukocytes ≥ 100/mm^3^ and CSF glucose ≤ 2.2 mmol/L. For patients meeting the laboratory criteria, we queried the “d_icd_procedures” table to identify whether they had undergone neurosurgery or invasive procedures. Only patients with documented neurosurgical interventions were retained for the external validation cohort. Among 248 patients who met the inclusion criteria, 77 patients were positive CSF etiological culture and 171 patients were negative CSF etiological culture. For patients with positive CSF etiological culture, 4 patients were under the age of 18, 10 without discharge diagnosis as BV/M, 14 were diagnosed as viral, fungal or tuberculous ventriculitis/meningitis, and 6 with incomplete records. For patients with negative CSF etiological culture, 137 patients were added/changed antibiotics, 10 were under the age of 18, and 9 with incomplete records. Finally, there were 43 confirmed BV/M patients and 15 confirmed non-BV/M patients ruled in the validation cohort-2 (Supplementary Figure S1). For all eligible patients, we extracted the relevant predictor variables required for applying our nomogram. This screening process ensured consistency between the external validation cohort and our training and internal validation cohorts.

### Screening for predictive variables

The variance inflation factors of the 20 variables were below 10, suggesting no interaction terms need to be considered (Supplementary Table S1). The multivariate logistic regression analysis showed that six variables were independent predictors related to BV/M, including blood lymphocyte proportions [*p* = 0.003, odds ratio (OR) 0.153, 95% confidence interval (CI) 0.042–0.505], CSF leukocytes [305–8,112/mm^3^ compared with <305/mm^3^ (*p* = 0.005, OR 9.499, 95% CI 2.117–49.370), ≥8,112/mm^3^ compared with <305/mm^3^ (*p* = 0.002, OR 18.678, 95% CI 3.280–125.357)], CSF erythrocytes (*p* < 0.001, OR 0.103, 95% CI 0.036–0.263), CSF neutrophil proportions (*p* = 0.004, OR 0.325, 95% CI 0.147–0.696), CSF glucose [1.14–1.94 mmol/L compared with 1.94–2.20 mmol/L (*p* = 0.010, OR 4.008, 95% CI 1.445–12.082), <1.14 mmol/L compared with 1.94–2.20 mmol/L (*p* < 0.001, OR 18.755, 95% CI 6.080–65.630)], and EVD (*p* = 0.001, OR 0.225, 95% CI 0.090–0.524) (Table 1).

**Table 1:** General characteristics of the patients and multivariate logistic regression analyses for screening predictors.

Variable		BV/M (*n* = 150) N (%)	Non-BV/M (*n* = 121) N (%)	*P*-value	OR	OR 95% CI	
Age (years)	18–45	44(29.3)	31(25.6)	Reference			
	45–60	63(42.0)	50(41.3)	0.464	0.698	0.263	1.823
	≥60	43(28.7)	40(33.1)	0.904	0.937	0.326	2.696
Sex	Female	44(29.3)	46(38.0)	Reference			
	Male	106(70.7)	75(62.0)	0.997	0.998	0.446	2.225
Surgical site	Non-occipital	101(67.3)	88(72.7)	Reference			
	Occipital	49(32.7)	33(27.3)	0.687	1.182	0.524	2.701
Admitting diagnosis	Non-hemorrhage	60(40.0)	45(37.2)	Reference			
	Hemorrhage	90(60.0)	76(62.8)	0.475	0.743	0.325	1.672
Temperature (°C)	<37.3	25(16.7)	30(24.8)	Reference			
	37.3–38.0	55(36.7)	49(40.5)	0.765	1.166	0.427	3.229
	38.1–39.0	54(36.0)	40(33.1)	0.922	0.952	0.350	2.558
	>39.0	16(10.7)	2(1.7)	0.028	17.643	1.710	286.631
CRP (mg/L)	<122	106(70.7)	104(86.0)	Reference			
	≥122	44(29.3)	17(14.0)	0.202	0.505	0.171	1.421
Blood leukocytes (×10^9^/L)	<13.12	107(71.3)	108(89.3)	Reference			
	≥13.12	43(28.7)	13(10.7)	0.625	0.771	0.269	2.204
Blood neutrophils (%)	<84.30	85(56.7)	96 (79.3)	Reference			
	≥84.30	65(43.3)	25(20.7)	0.395	1.708	0.502	5.990
Blood lymphocytes (%)	<9.05	71(47.3)	26(21.5)	Reference			
	≥9.05	79(52.7)	95(78.5)	0.003	0.153	0.042	0.505
Blood PCT (ng/ml)	<0.46	92(61.3)	104(86.0)	Reference			
	≥0.46	58(38.7)	17(14.0)	0.025	0.343	0.130	0.857
Blood lactate (mmol/L)	<1.14	25(16.7)	41(33.9)	Reference			
	≥1.14	125(83.3)	80(66.1)	0.671	0.813	0.310	2.122
CSF leukocytes (/mm^3^)	<305	5(3.3)	13(10.7)	Reference			
	305–8,112	96(64.0)	88(72.7)	0.005	9.499	2.117	49.370
	≥8,112	49(32.7)	20(16.5)	0.002	18.678	3.280	125.357
CSF erythrocytes (/mm^3^)	<37,250	118(78.7)	55(45.5)	Reference			
	≥37,250	32(21.3)	66(54.5)	<0.001	0.103	0.036	0.263
CSF neutrophils (%)	<88.2	57(38.0)	82(67.8)	Reference			
	≥88.2	93(62.0)	39(32.2)	0.004	0.325	0.147	0.696
CSF glucose (mmol/L)	1.94–2.20	11(7.3)	40(33.1)	Reference			
	1.14–1.94	48(32.0)	51(42.1)	0.010	4.008	1.445	12.082
	<1.14	91(60.7)	30(24.8)	<0.001	18.755	6.080	65.630
CSF proteins (mg/L)	<2,215	34(22.7)	47 (38.8)	Reference			
	≥2,215	116(77.3)	74(61.2)	0.656	1.213	0.519	2.878
Concurrent infection	Yes	97(64.7)	48(39.7)	Reference			
	No	53(35.3)	73(60.3)	0.049	0.456	0.206	0.990
GCS	15	8(5.3)	11(9.1)	Reference			
	12–14	14(9.3)	3(2.5)	0.857	1.215	0.154	11.466
	9–11	17(11.3)	11(9.1)	0.705	0.716	0.129	4.211
	2–8	111(74.0)	96(79.3)	0.351	0.503	0.120	2.237
EVD	Yes	101 (67.3)	57(47.1)	Reference			
	No	49(32.7)	64(52.9)	0.001	0.225	0.090	0.524
Transparency of CSF	Clear	19(12.7)	13(10.7)	Reference			
	Slightly muddy	46(30.7)	33(27.3)	0.554	0.673	0.177	2.492
	Turbid	85(56.7)	75(62.0)	0.275	0.471	0.119	1.807

BV/M, Bacterial ventriculitis/meningitis; OR, Odds ratio; CI, Confidence interval; CRP, C-reactive protein; PCT, Procalcitonin; CSF, Cerebrospinal fluid; GCS, Glasgow coma scale; EVD, External ventricular drainage.

### Developing the risk prediction model

**Figure 2:**
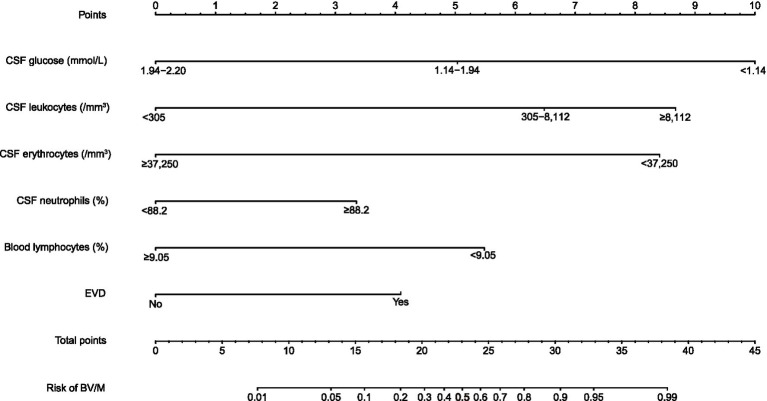
Nomogram constructed by six variables for the risk of post-neurosurgical bacterial ventriculitis/meningitis. CSF, Cerebrospinal fluid; EVD, external ventricular drainage; BV/M, bacterial ventriculitis/meningitis.

The above six variables were integrated into the nomogram (Figure 2). The risk level was divided into the low-risk group (<0.5) and high-risk group (≥0.5). For a specific patient, the higher total points indicated the higher risk of BV/M. For example, if a patient with CSF glucose of 1.10 mmol/L (<1.14 mmol/L), CSF leukocytes of 7,000/mm^3^ (305–8,112/mm^3^), CSF erythrocytes of 15,000/mm^3^ (<37,250/mm^3^), CSF neutrophil proportions of 80% (<88.2%), blood lymphocyte proportions of 10.00% (≥9.05%), and EVD, the corresponding score of this patient would be approximately 10, 6.4, 8.3, 0, 0, and 4.2, respectively. Accordingly, the total score was about 28.9, and the risk of BV/M was 0.85, thus indicating a relatively high probability of suffering from BV/M.

### Assessing the accuracy of the prediction model

**Figure 3:**
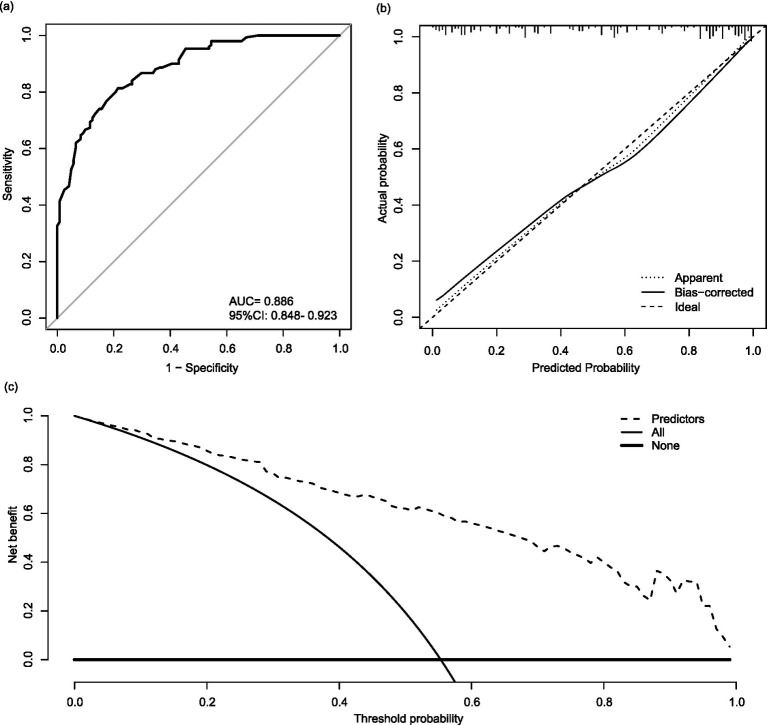
Evaluation of the nomogram in the training cohort. **(a)** Receiver operating characteristic curve. **(b)** Calibration curve. **(c)** Decision curve analysis. AUC, Area under the receiver operating characteristic curve; CI, confidence interval.

First, the predictive accuracy of the nomogram in the training cohort was tested, the Hosmer-Lemeshow test was used, it was found that the nomogram possessed a good fit (*p* = 0.471), indicating no significant difference existed between the observed and predicted outcomes. The AUC of the nomogram arrived 0.886 (Figure 3a), while a value of AUC close to 1.000 reflected a good accuracy. The calibration curve displayed that apparent curve and bias-corrected curve approached the ideal diagonal line (Figure 3b), which suggested the predominant consistency of the nomogram. Moreover, the DCA demonstrated a significant net benefit as evidenced by the predicted curve was away from other lines (Figure 3c).

**Figure 4:**
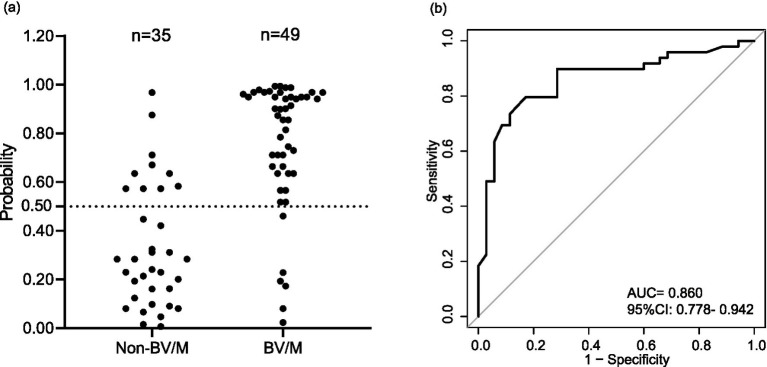
Evaluation of the nomogram in the validation cohort-1. **(a)** Distribution of probability. **(b)** Receiver operating characteristic curve. BV/M, Bacterial ventriculitis/meningitis; AUC, Area under the receiver operating characteristic curve; CI, confidence interval.

Second, the predictive accuracy of the nomogram was tested by the validation cohort including 84 patients (Figure 4). In this work, the probability value of 0.5, a threshold employed to differentiate the high-risk and low-risk post-neurosurgical BV/M, was set for avoiding false-negative errors as much as possible. Accordingly, among 49 patients with confirmed BV/M, 6 patients were mistaken as non-BV/M, while 10 patient was mistaken as BV/M among 35 patients with confirmed non-BV/M. The AUC of the nomogram in the validation cohort arrived 0.860.

**Figure 5:**
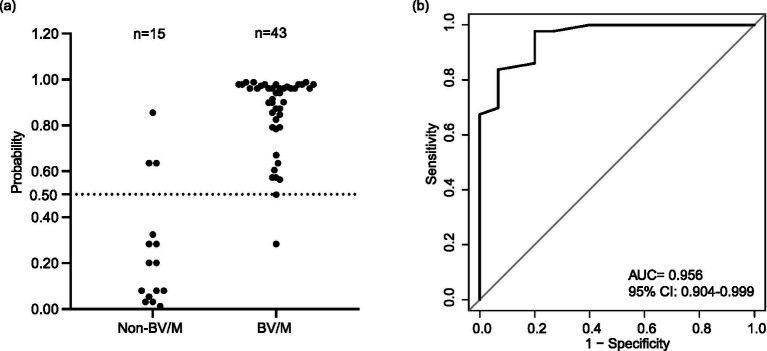
Evaluation of the nomogram in the validation cohort-2. **(a)** Distribution of probability. **(b)** Receiver operating characteristic curve. BV/M, Bacterial ventriculitis/meningitis; AUC, Area under the receiver operating characteristic curve; CI, Confidence interval.

Finally, the external validation for the nomogram was also assessed by 58 patients from the MIMIC-III and MIMIC-IV databases, and the risk of BV/M for each patient was shown in Figure 5a and Supplementary Table S2. Among 43 patients with confirmed BV/M, 2 patients were mistaken as non-BV/M, while 3 patient was mistaken as BV/M among 15 patients with confirmed non-BV/M. Besides, a high AUC value of 0.956 was also observed (Figure 5b).

Furthermore, other parameters of the efficiency verification also indicated that our model might be a potentially available tool for early diagnosis of post-neurosurgical BV/M. In training datasets, the established early diagnosis model possessed an accuracy of 0.790 (0.736–0.837), a sensitivity of 0.827 (95% CI: 0.756–0.884), a specificity of 0.744 (95% CI: 0.657–0.819), a positive predictive value (PPV) of 0.800 (95% CI: 0.728–0.860), a negative predictive value (NPV) of 0.776 (0.689–0.848). In validation cohort-1, the model possessed an accuracy of 0.810 (0.709–0.887), a sensitivity of 0.878 (95% CI: 0.752–0.954), a specificity of 0.714 (95% CI: 0.530–0.854), a PPV of 0.811 (95% CI: 0.680–0.906), a NPV of 0.807 (0.625–0.926). In validation cohort-2, the model possessed an accuracy of 0.914 (0.810–0.971), a sensitivity of 0.954 (95% CI: 0.842–0.994), a specificity of 0.800 (95% CI: 0.519–0.957), a PPV of 0.932 (95% CI: 0.813–0.986), a NPV of 0.857 (0.572–0.982) (Table 2).

**Table 2:** Performance of the nomogram with six variables in the training cohort, validation cohort-1, and validation cohort-2.

	Training cohort	Validation cohort-1	Validation cohort-2
Accuracy (95% CI)	0.790 (0.736–0.837)	0.810 (0.709–0.887)	0.914 (0.810–0.971)
Sensitivity (95% CI)	0.827 (0.756–0.884)	0.878 (0.752–0.954)	0.954 (0.842–0.994)
Specificity (95% CI)	0.744 (0.657–0.819)	0.714 (0.530–0.854)	0.800 (0.519–0.957)
PPV (95% CI)	0.800 (0.728–0.860)	0.811 (0.680–0.906)	0.932 (0.813–0.986)
NPV (95% CI)	0.776 (0.689–0.848)	0.807 (0.625–0.926)	0.857 (0.572–0.982)

AUC, Area under the receiver operating characteristic curve; CI, Confidence interval; PPV, positive predictive value; NPV, negative predictive value. Training cohort, 271 patients from the three hospitals; Validation cohort-1, 84 patients from the three hospitals; Validation cohort-2, 58 patients from MIMIC-III/IV databases.

These together indicated that our established nomogram possessed a high discriminative power to predict the risk of post-neurosurgical BV/M in suspected BV/M patients with leukocytic pleocytosis and hypoglycorrhachia.

## Discussion

Post-neurosurgical BV/M is a potentially catastrophic infectious disease associated with substantial mortality and a risk of permanent disability in survivors. Early antibiotic prescription is a crucial intervention to reduce mortality in patients with BV/M characterized by leukocytic pleocytosis and hypoglycorrhachia. However, not all patients, who met leukocytic pleocytosis and hypoglycorrhachia, should be diagnosed as BV/M. In this retrospective multicenter study, 121 of 711 patients with leukocytic pleocytosis and hypoglycorrhachia were finally diagnosed as non-BV/M based on the follow-up. These strongly suggested that unnecessary antibiotic usage did exist, which only contributed to the development of antimicrobial resistance and the risk of adverse drug reactions.

In this work, we employ established statistical methods, the original contribution is the development and rigorous validation of a practical tool that meaningfully outperforms simpler approaches. Its ability to provide granular risk stratification addresses a specific gap in current management for BM/V, facilitating a step toward personalized medicine. The risk assessment scoring model based on easily available clinical and laboratory parameters to differentiate the high-risk and low-risk post-neurosurgical BV/M in suspected BV/M patients with leukocytic pleocytosis (above 100/mm^3^) and hypoglycorrhachia (below 2.2 mmol/L).

This model included six variables: CSF glucose, CSF leukocytes, CSF erythrocytes, CSF neutrophil proportions, blood lymphocyte proportions, and EVD, which were filtered out from 20 parameters. The risk of BV/M for suspected patients could be predicted according to the score calculated on the six parameters, and was divided into the low-risk and the high-risk group. In our view, antibiotics should be immediately administered for high-risk patients, and for the low-risk, analysis the changes of parameters by increasing CSF examination frequency was recommended prior to antibiotics treatment.

It is well-accepted that leukocytic pleocytosis and hypoglycorrhachia are important reference parameters for the diagnosis of BV/M. Classic abnormalities of CSF composition in BV/M are low glucose concentrations and elevated leukocyte levels (7). Wilhelmina et al. found that patients with bacterial meningitis usually had low CSF glucose values referred to below 2.2 or 2.5 mmol/L combined with other abnormal CSF parameters (14). Welch et al. used CSF glucose level < 2.8 mmol/L as a diagnostic indicator for post-neurosurgical bacterial meningitis, and found that the concentration of CSF glucose would gradually rise when the bacterial meningitis was effectively controlled (15). Thijs et al. reported that CSF leukocyte count > 2,000/mm^3^ was a risk factor for bacterial meningitis (16). CSF leukocyte counts would decrease when the post-neurosurgical bacterial meningitis was effectively controlled (17). It was believed that an elevated number of leukocytes in the CSF with a neutrophilic predominance was highly suggestive of BV/M (15, 18). The higher levels of neutrophil percentage predicted the increased risk of many infectious diseases (19–21). In this study, the percentage of CSF neutrophile granulocytes was increased in BV/M patients.

Olga et al. demonstrated that aneurysmal subarachnoid hemorrhage was one of the risk variables for nosocomial meningitis but did not show a relationship between CSF erythrocytes and nosocomial meningitis (22). Polycythemia of CSF and hypoglycorrhachia may overlap in several conditions such as hemorrhage. In this study, there was a negative correlation between CSF erythrocytes and the occurrence of BV/M, and the risk was higher when CSF erythrocytes were under 37,250/mm^3^. One of the possible explanations for this negative correlation was that the anaerobic glycolysis of CSF erythrocytes required the consumption of CSF glucose. It was often occurred that the suspected BV/M patients with a mass of CSF erythrocytes eventually received a reversed diagnosis because of low CSF glucose levels (23, 24). For the first time, the CSF erythrocytes was used as a diagnostic parameter for BV/M.

The etiology of lymphopenia encompasses a range of factors, including therapy postoperative stress, systemic infection, autoimmune disorders, pharmacological interventions, and exposure to radiation (25–28). Gu et al. reported that the blood neutrophil-lymphocyte ratio was associated with central nervous system infection and the higher this ratio, that was, more neutrophils and fewer lymphocytes, the more attention should be paid to the possible infection (29, 30). Sepsis-induced lymphopenia is gradually being recognized as an essential factor in the prognosis of sepsis. Multiple studies have repeatedly observed a significant decrease in circulating lymphocyte count (31–35) which imply that lymphopenia is reliable for forecasting short-term and long-term patient outcomes. However, the actual mechanisms of the association between low lymphocyte count and poor prognosis are unclear. It is generally believed that the low lymphocyte count may be associated with a preexisting immunosuppressed condition, suggesting that the host tends to have an inadequate immunological reaction (27, 36, 37). And stress hormones (e.g., cortisol) and inflammatory cytokines (e.g., IL-6, TNF-*α*) released during the perioperative period or due to underlying disease can directly induce lymphocyte apoptosis and suppress proliferation, contributing to the observed low proportion (38). In this work, patients were classified into the high percentage of lymphocytes and the low percentage of lymphocytes group with a cutoff value of 9.05%. And we need to dig deeper into the causes of lymphocytopenia, such as postoperative stress, systemic infection, and glucocorticoids usage in future research.

The presence of EVD was another risk factor for bacterial ventriculitis and the incidence of EVD-related central nervous system infection might reach up to 36% (36). Rogier et al. reported that the duration of EVD placement, CSF sampling frequency withdrawal from the EVD, manipulation, and site leakage affect the occurrence of bacterial meningitis (37). In our work, 67% of confirmed BV/M patients were with EVD.

The limitation of this work was some important clinical and laboratory parameters, such as CSF lactate, CSF procalcitonin, CSF leakage, and CSF/blood glucose ratio etc., were excluded in the established nomogram due to the lack of data in our enrolled patients, which may lead to false negative or positive errors. And we used a strict negative control group, which reduced the universality of the model. We hope that new biomarkers will emerge in the future to distinguish infected patients among those with negative cultures and a changed treatment. Moreover, considering the usability of nomogram, we will develop an online calculator later.

## Conclusion

In summary, we established and validated a nomogram to predict the risk of BV/M in suspected post-neurosurgical patients with abnormal CSF leukocytes (above 100/mm^3^) and glucose value (below 2.2 mmol/L) based on six easily available indexes, and thus provide a suggested protocol, that is, antibiotics should be immediately administered for high-risk patients, while for the low-risk, analysis the changes of parameters by increasing CSF examination frequency was recommended prior to antibiotics treatment.

## Data Availability

The data analyzed in this study is subject to the following licenses/restrictions: Patients data from three hospitals can be obtained from author and patients from MIMIC-III and MIMIC-IV can be obtained from common data base. Requests to access these datasets should be directed to minni@tongji.edu.cn.
