# Supplementary material for: Development and validation of a nomogram for suspected post-neurosurgical bacterial ventriculitis/meningitis
Source: Front Med (Lausanne). 2026 Mar 18;13:1790954. doi: 10.3389/fmed.2026.1790954 (PMC13039033; doi:10.3389/fmed.2026.1790954)

# Supplementary material

## Development and validation of a nomogram for suspected post-neurosurgical bacterial ventriculitis/meningitis

**Table S1: Variance inflation factors of each factor.**

| Variable | Variance inflation factors |
|---|---|
| Age | 1.120 |
| Sex | 1.116 |
| Surgical site | 1.094 |
| Admitting diagnosis | 1.147 |
| Temperature | 1.105 |
| CRP | 1.105 |
| Blood leukocytes | 1.137 |
| Blood neutrophil proportions | 1.601 |
| Blood lymphocyte proportions | 1.634 |
| Blood PCT | 1.085 |
| Blood lactate | 1.218 |
| CSF leukocytes | 1.178 |
| CSF erythrocytes | 1.399 |
| CSF neutrophil proportions | 1.111 |
| CSF glucose | 1.127 |
| CSF proteins | 1.131 |
| Concurrent infection | 1.124 |
| GCS | 1.071 |
| EVD | 1.234 |
| Transparency of the CSF | 1.263 |

CRP, C-reactive protein; PCT, Procalcitonin; CSF, Cerebrospinal fluid; GCS, Glasgow coma scale; EVD, External ventricular drainage.

**Table S2. The external validation cohort from MIMIC-III and MIMIC-IV database**

| MIMIC-III | | | | MIMIC-IV | | | |
|---|---|---|---|---|---|---|---|
| Subject_ID | Outcome | Score | Probability | Subject_ID | Outcome | Score | Probability |
| 74696 | BV/M | 37.819 | 0.988 | 17681429 | BV/M | 37.819 | 0.988 |
| 62183 | BV/M | 35.920 | 0.979 | 15188685 | BV/M | 37.819 | 0.988 |
| 49603 | BV/M | 35.920 | 0.979 | 17336353 | BV/M | 35.920 | 0.979 |
| 14486 | BV/M | 35.920 | 0.979 | 11488867 | BV/M | 35.920 | 0.979 |
| 10316 | BV/M | 35.920 | 0.979 | 11317682 | BV/M | 35.920 | 0.979 |
| 27688 | BV/M | 34.468 | 0.968 | 18855794 | BV/M | 35.043 | 0.973 |
| 87336 | BV/M | 33.728 | 0.961 | 18654207 | BV/M | 33.728 | 0.961 |
| 59731 | BV/M | 33.728 | 0.961 | 15151307 | BV/M | 33.728 | 0.961 |
| 57663 | BV/M | 33.728 | 0.961 | 13806563 | BV/M | 33.728 | 0.961 |
| 53411 | BV/M | 33.728 | 0.961 | 17416494 | BV/M | 32.334 | 0.941 |
| 23523 | BV/M | 33.728 | 0.961 | 14500728 | BV/M | 30.953 | 0.914 |
| 7683 | BV/M | 33.728 | 0.961 | 16751740 | BV/M | 29.501 | 0.873 |
| 22421 | BV/M | 32.334 | 0.941 | 16607081 | BV/M | 29.501 | 0.873 |
| 14958 | BV/M | 30.436 | 0.901 | 15114055 | non-BV/M | 28.983 | 0.855 |
| 24369 | BV/M | 30.377 | 0.900 | 11343484 | BV/M | 28.983 | 0.855 |
| 13599 | BV/M | 30.377 | 0.900 | 11320864 | BV/M | 28.761 | 0.847 |
| 97158 | BV/M | 28.244 | 0.826 | 12574016 | BV/M | 27.509 | 0.792 |
| 45703 | BV/M | 27.509 | 0.792 | 18919151 | BV/M | 27.368 | 0.785 |
| 11093 | BV/M | 25.410 | 0.670 | 15529775 | non-BV/M | 24.893 | 0.635 |
| 81195 | non-BV/M | 24.893 | 0.635 | 14620541 | BV/M | 24.468 | 0.605 |
| 16533 | BV/M | 24.893 | 0.635 | 19342961 | BV/M | 24.017 | 0.573 |
| 17151 | BV/M | 19.926 | 0.283 | 10731982 | BV/M | 24.017 | 0.573 |
| 9443 | non-BV/M | 19.926 | 0.283 | 15729731 | BV/M | 23.895 | 0.564 |
| 94422 | non-BV/M | 14.867 | 0.080 | 16890177 | BV/M | 23.018 | 0.499 |
| 43446 | non-BV/M | 11.516 | 0.031 | 18908641 | non-BV/M | 20.573 | 0.324 |
| | | | | 17299746 | non-BV/M | 19.926 | 0.283 |
| | | | | 16477516 | non-BV/M | 18.410 | 0.201 |
| | | | | 14863189 | non-BV/M | 18.410 | 0.201 |
| | | | | 19513478 | non-BV/M | 14.867 | 0.080 |
| | | | | 18047173 | non-BV/M | 14.867 | 0.080 |
| | | | | 18589545 | non-BV/M | 13.444 | 0.054 |
| | | | | 17906440 | non-BV/M | 11.516 | 0.031 |
| | | | | 11822564 | non-BV/M | 8.410 | 0.013 |

BV/M, Bacterial ventriculitis/meningitis.

**Figure S1. Flow chart for patient selection in the validation cohort.** CSF, Cerebrospinal fluid; BV/M, Bacterial ventriculitis/meningitis.

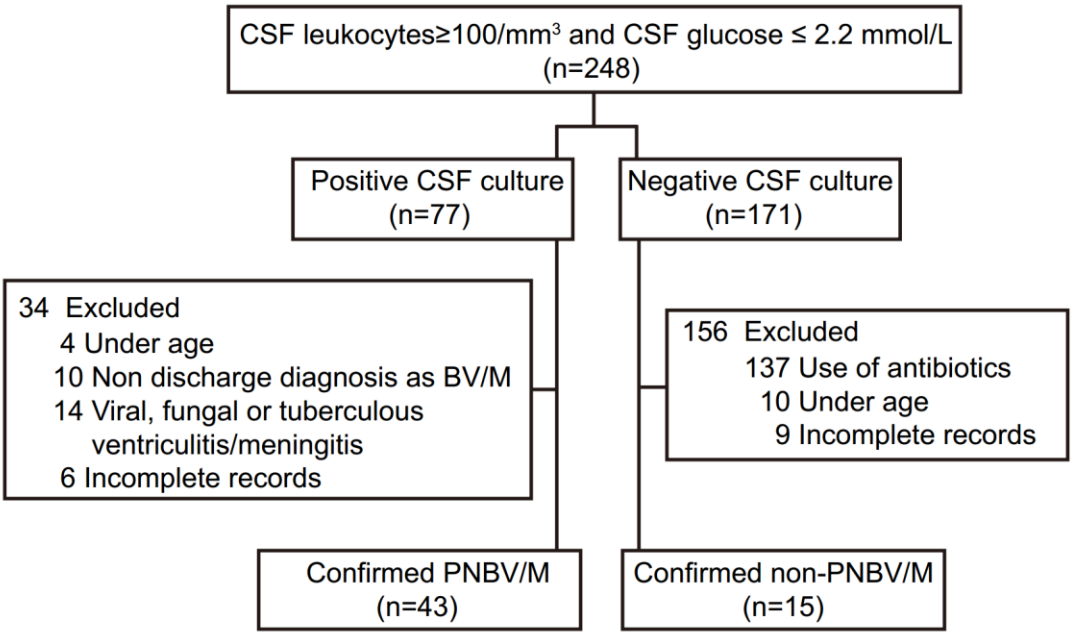

Supplement: Supplementary file 1 [file Data_sheet_1.pdf]
